# Fundamental Movement Skills Are More than Run, Throw and Catch: The Role of Stability Skills

**DOI:** 10.1371/journal.pone.0140224

**Published:** 2015-10-15

**Authors:** James R. Rudd, Lisa M. Barnett, Michael L. Butson, Damian Farrow, Jason Berry, Remco C. J. Polman

**Affiliations:** 1 Institute of Sport Exercise and Active Living, Victoria University, Melbourne, Australia; 2 School of Health and Social Development, Deakin University, Melbourne, Australia; 3 Movement Science, Australian Institute of Sport, Canberra, Australia; 4 Psychology Department, Bournemouth University, Bournemouth, United Kingdom; University of Milan, ITALY

## Abstract

**Introduction:**

In motor development literature fundamental movement skills are divided into three constructs: locomotive, object control and stability skills. Most fundamental movement skills research has focused on children’s competency in locomotor and object control skills. The first aim of this study was to validate a test battery to assess the construct of stability skills, in children aged 6 to 10 (*M* age = 8.2, SD = 1.2). Secondly we assessed how the stability skills construct fitted into a model of fundamental movement skill.

**Method:**

The Delphi method was used to select the stability skill battery. Confirmatory factor analysis (CFA) was used to assess if the skills loaded onto the same construct and a new model of FMS was developed using structural equation modelling.

**Results:**

Three postural control tasks were selected (the log roll, rock and back support) because they had good face and content validity. These skills also demonstrated good predictive validity with gymnasts scoring significantly better than children without gymnastic training and children from a high SES school performing better than those from a mid and low SES schools and the mid SES children scored better than the low SES children (all p < .05). Inter rater reliability tests were excellent for all three skills (ICC = 0.81, 0.87, 0.87) as was test re-test reliability (ICC 0.87–0.95). CFA provided good construct validity, and structural equation modelling revealed stability skills to be an independent factor in an overall FMS model which included locomotor (r = .88), object control (r = .76) and stability skills (r = .81).

**Discussion:**

This study provides a rationale for the inclusion of stability skills in FMS assessment. The stability skills could be used alongside other FMS assessment tools to provide a holistic assessment of children’s fundamental movement skills.

## Introduction

The ability to perform various fundamental movement skills (FMS) (e.g., running, catching, hopping, throwing) in a consistent and proficient manner, is often defined as movement competence [1,2]. High levels of FMS competence in childhood are related to a number of health and physical activity outcomes [3]. Children who possess high FMS levels have a greater chance of maintaining good health, are more likely to participate in physical activity and possess better fitness in later life [4,5].

Yet Australian research has demonstrated low and decreasing levels of FMS [6–8]. This may be due to many children missing out on the foundations of movement which were routinely developed by children in previous generations through incidental physical activity. Australia has seen a 42% decline in active transport between 1971 and 2013 and children’s top ten preferred play spaces have seen a marked transition from outdoors to indoors between 1950 to 2000 [9].

Gallahue, Ozmun and Goodway [1] state that there are three constructs which make up FMS: locomotor (run, hop, jump, slide, gallop, leap); object control (strike, dribble, kick, throw, underarm roll, catch); and stability skills (non locomotor skills such as body rolling, bending, and twisting). Object and locomotor skills have been widely evaluated in children’s FMS development, [3,5,8,10,11]. The same cannot be said for stability skills which have been described as the most basic skills within the FMS family [1].

Stability skills can be defined as the ability to sense a shift in the relationship of the body parts that alter one’s balance, as well as the ability to adjust rapidly and accurately to these changes with the appropriate compensating movements [1]. The system responsible for the ability to maintain balance and sense shifts in balance is generally termed postural control and enables the body’s positioning in space for the dual purposes of stability and orientation. Postural stability refers to the ability to maintain, achieve or restore a specific state of balance, whilst postural orientation is the competence to maintain an appropriate relationship between the body and the environment for a task [12].

Few studies have investigated the relationship of balance to other FMS. Results of such studies highlight that balance is task specific and a dynamic process and that one specific type of (static) balance test is potentially an unreliable measure for stability skills which are underpinned by a child’s postural control system. For instance, Ulrich and Ulrich [13] showed that the composite balance test from the Bruininks-Oseretsky test of motor proficiency in 3–5 year olds, significantly predicted a qualitative rating of hopping, jumping and striking proficiency, but not other FMS. Ulrich and Ulrich speculated that the composite score for balance may be too insensitive to assess the specific types of balance control required in other FMS. Chew-Bullock et al. [14] found a significant correlation between single leg balance and kicking accuracy but not kicking velocity. These findings are consistent with the notion that when kicking for velocity, the center of gravity will be outside of the body, to utilise momentum so as to increase power, making it unlikely that maintaining static balance would be of importance (see Butterfield & Loovis [15] for similar results).

The Lower Quarter Y Balance Test (YBT-LQ) has been used to assess a similar concept to stability skills in children. This dynamic product based assessment tool requires children to maintain a single-leg balance and reach as far as possible with the contralateral leg in the anterior, posteromedial and posterolateral directions. The YBT-LQ has been shown to have good inter-rater and retest reliability; although predictive validity could not be established [16]. It was suggested that other factors need to be considered alongside chronological age when assessing predictive validity such as somatotype, muscular strength and habitual physical activity [16]. For example, there is evidence to suggest that weight status (obesity) is associated with poorer FMS [3]. Furthermore it was suggested that environmental factors may have caused some children to develop more efficient movement strategies resulting in higher stability scores [16]. This is supported by other studies which found that socioeconomic status (SES) influences maturational development [17,18], weight status and the acquisition of FMS [19,20]. Despite this, no specific research has examined how SES affects stability skill proficiency.

Participation in gymnastics is thought to promote improvements in the performance of postural control of younger children through the use of sensory cues inherent in the execution of gymnastic skills. Garcia, Barela, Viana and Barela [21] found significant improvements in bipedal (static upright two foot stance) postural control in 5–7 year old gymnasts compared to non-gymnasts. However, the authors suggested it would be useful to examine further different postural control stances that place higher demands on children’s postural control system [21].

The first aim of this study was to validate a test battery to assess stability skills in children aged 6 to 10 years old in order to measure the development of the underpinning sub-domains of postural control system, orientation and stability. The second aim of this study was to assess where stability skills fit into a FMS model which includes locomotive and object control skills. We also investigated the influence of SES as a predictor of stability skills development as well as the possible influence of (grip) strength and body mass index (BMI) [16].

## Method

The method is divided into three parts: Part one sets out the procedure for developing the stability skills assessment tool to measure the face and content validity of the test battery. Part two reports the methods used to assess predictive validity and inter and retest reliability. Part three explains the methods used to assess how stability fits into a FMS model, which involved two steps: a) confirmatory factor analysis to determine if the three stability skills examined load into the stability construct and b) structural equation modelling to develop a complete model of FMS which includes stability, locomotor and object control skills.

### Part One: developing Stability Skills Assessment Tool

#### Stability skill test protocol development

The development of the postural control test protocols was guided by the Delphi approach [22]. This method makes use of the opinions of a panel of experts through a series of carefully developed stages to create consensus. In particular, a panel of experts was used to determine face (measures what it is supposed to measure) and content (how essential test and its components are) validity [23].

#### Face validity

Four experts (three academic experts in human movement and skill acquisition and one physical education teacher) identified movement skills demanding postural control. Due to the relationship between superior postural control and gymnastics [21], the experts also reviewed 32 gymnastics skills (taken from the Gym Mix gymnastics for all national program) for potential inclusion in the postural control assessment tool. These skills were then ranked according to the demands they place on the two subdomains of the postural control system and the method by which this could be assessed.

In the first iteration, nine skills were identified: cartwheel, handstand, arabesque (a body position in which one stands on one leg with the other leg extended behind the body, both legs should be held straight), forward roll, backward roll, rock (a training method for the forward roll), front support, back support (a static wedge shape with arms straight and legs straight and together) and log roll (a sideways roll with arm and legs straight and slightly raised off the ground).

The second iteration assessed the feasibility of the skills as an assessment tool in a school setting, resulting in four skills being deemed unsuitable because of safety concerns (cartwheel, handstand, forward roll and backward roll) and one skill (arabesque) being similar to the YBT-LQ single leg balance.

This left four skills: rock, log roll, front support and back support. The front and back support are very similar skills so it was decided only one needed to be included. We selected the back support task as it was reasoned that it would be more challenging due to it being a more unnatural position for the body to hold and therefore would require higher torso strength and postural stability.

As each of these skills measure different aspects of postural control, i.e. the rock has high orientation demands, the back support requires high whole body stability and the log roll requires both postural control and stability, it was believed that when combined they would provide a holistic picture of participants’ postural control ability and as such be a good measure for the stability skills construct.

#### Content validity

A process-oriented assessment was developed for the three gymnastics skills similar to other FMS test batteries (e.g., Test of Gross Motor Development-2) [24]. This was achieved by filming (JVC GY/HM100E) an elite gymnast from two angles (90 degrees and front on) executing the three skills. The same team of experts involved in the development of face validity analysed each skill in slow-motion and agreed upon the key components for successful execution for each skill. This was the first iteration of a scoring system for each skill which enabled an assessor to determine if key components were present or absent.

Following this, nine experts (five academics, two physical education teachers and two state level gymnastics coaches) were invited to assess the skill components. To be included on the expert panel researchers had to have published papers internationally in the areas within or related to movement sciences; teachers had to have taught physical education or coached gymnastics to primary school aged school children; and gymnastic coaches needed to have advanced coach accreditation and be currently coaching.

Using email, each panel member was provided with the assessment elements and procedure of the rock, log roll and back support and were asked to examine whether the identified components were the key elements for successful skill execution and to rank each of the two postural control demands (orientation and stability) of each skill on a Likert scale (1 = low; 5 = high). All panel members provided extensive feedback which centered around three themes: 1) the wording of the components was overly scientific for mainstream use; 2) separate components overlapped in the same skill; 3) two of the three skills were deemed to be eliciting low levels of postural orientation or stability demands. Based on this feedback a number of changes were made.

**Fig 1:**
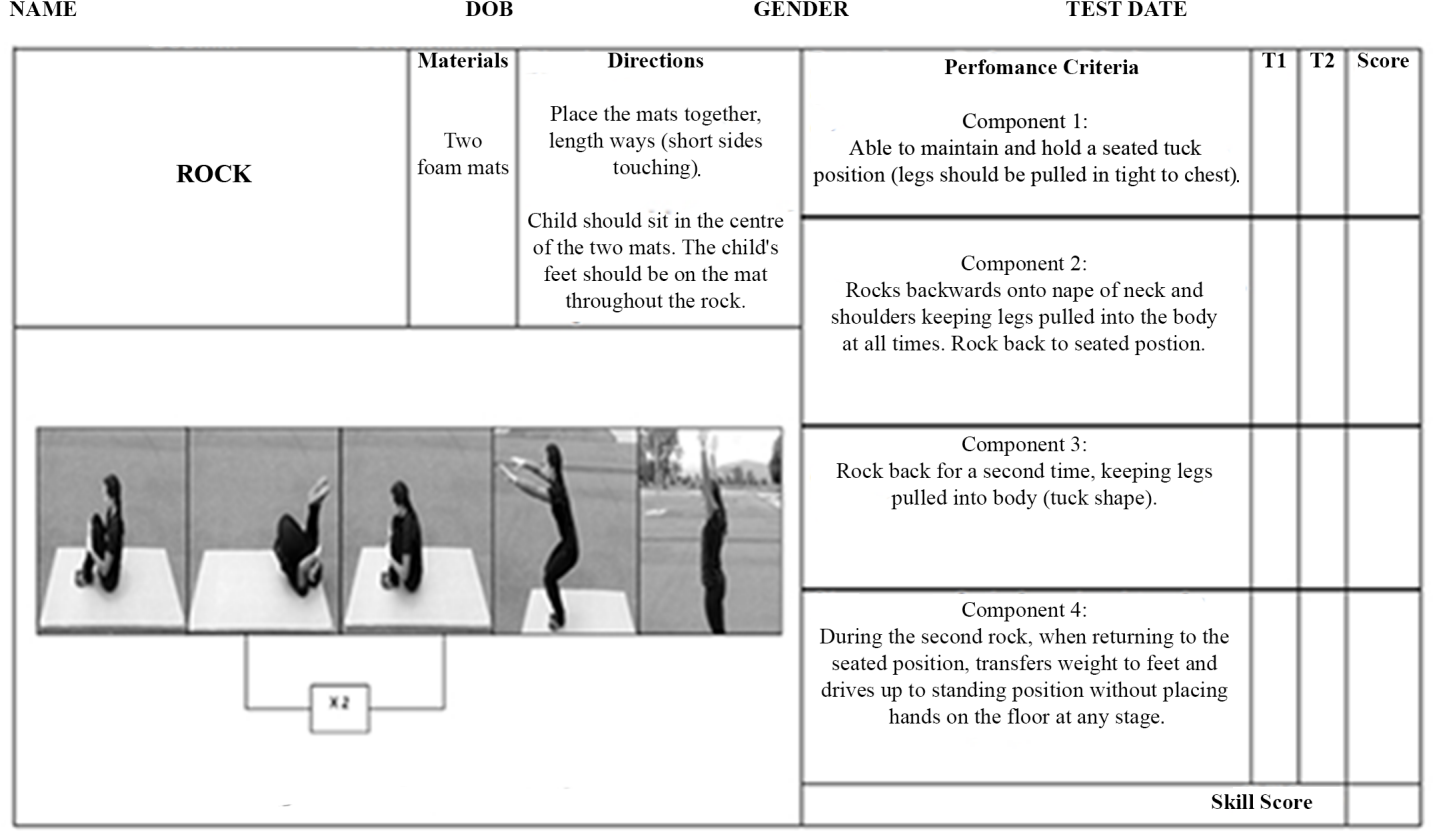
Rock scoresheet.

Rock: Changes were made to the protocol whereby the participants were required to complete two rocks and then come to a stand in a single motion to enhance the postural orientation demands of the skill. This was broken down into four components (Fig 1).

**Fig 2:**
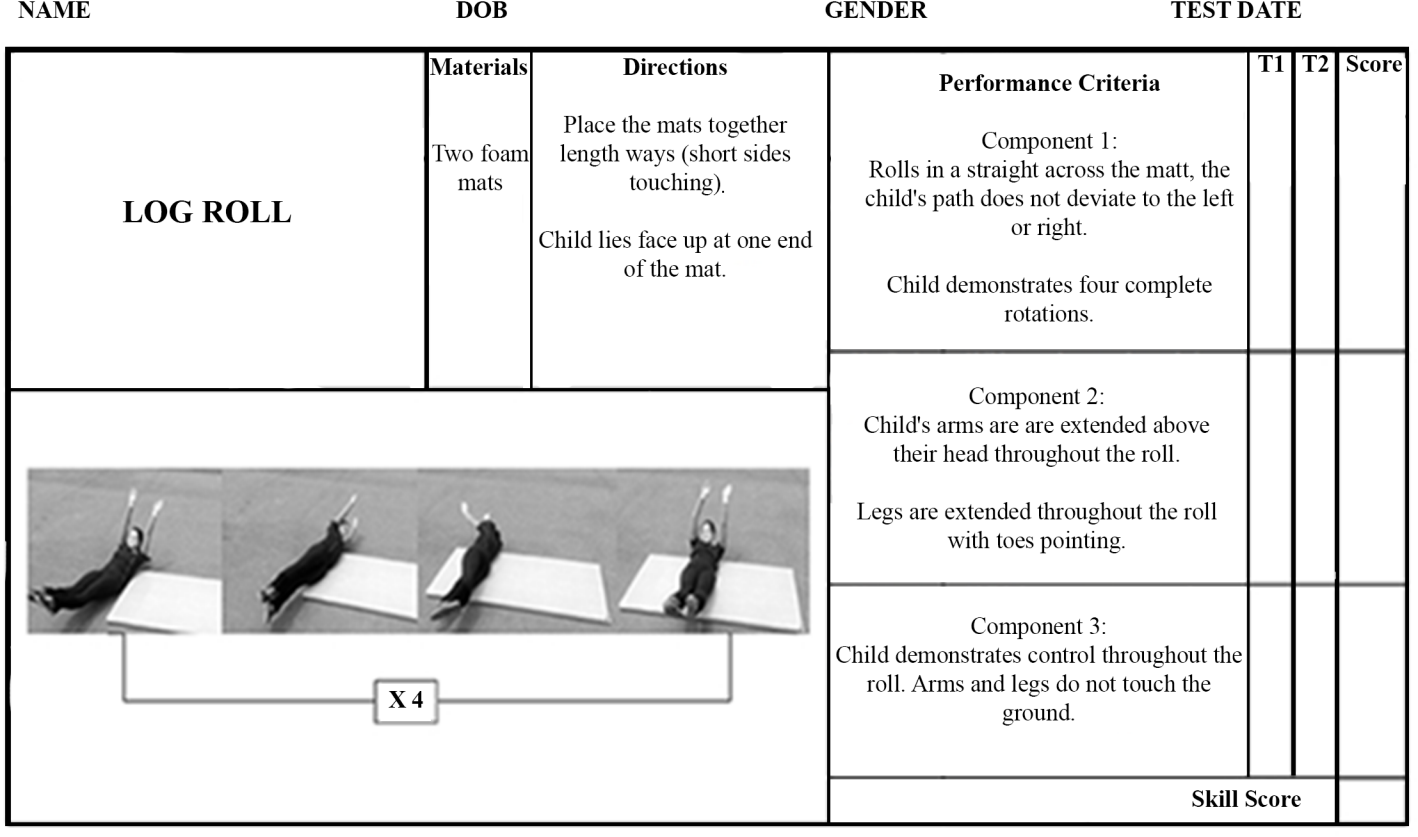
Log-roll scoresheet.

Log Roll: The log-roll protocol underwent the least revisions as it was felt it was the most demanding of skills, requiring orientation to roll in a straight line and stability to keep legs extended and slightly off the ground. The skill was condensed into three components (Fig 2).

**Fig 3:**
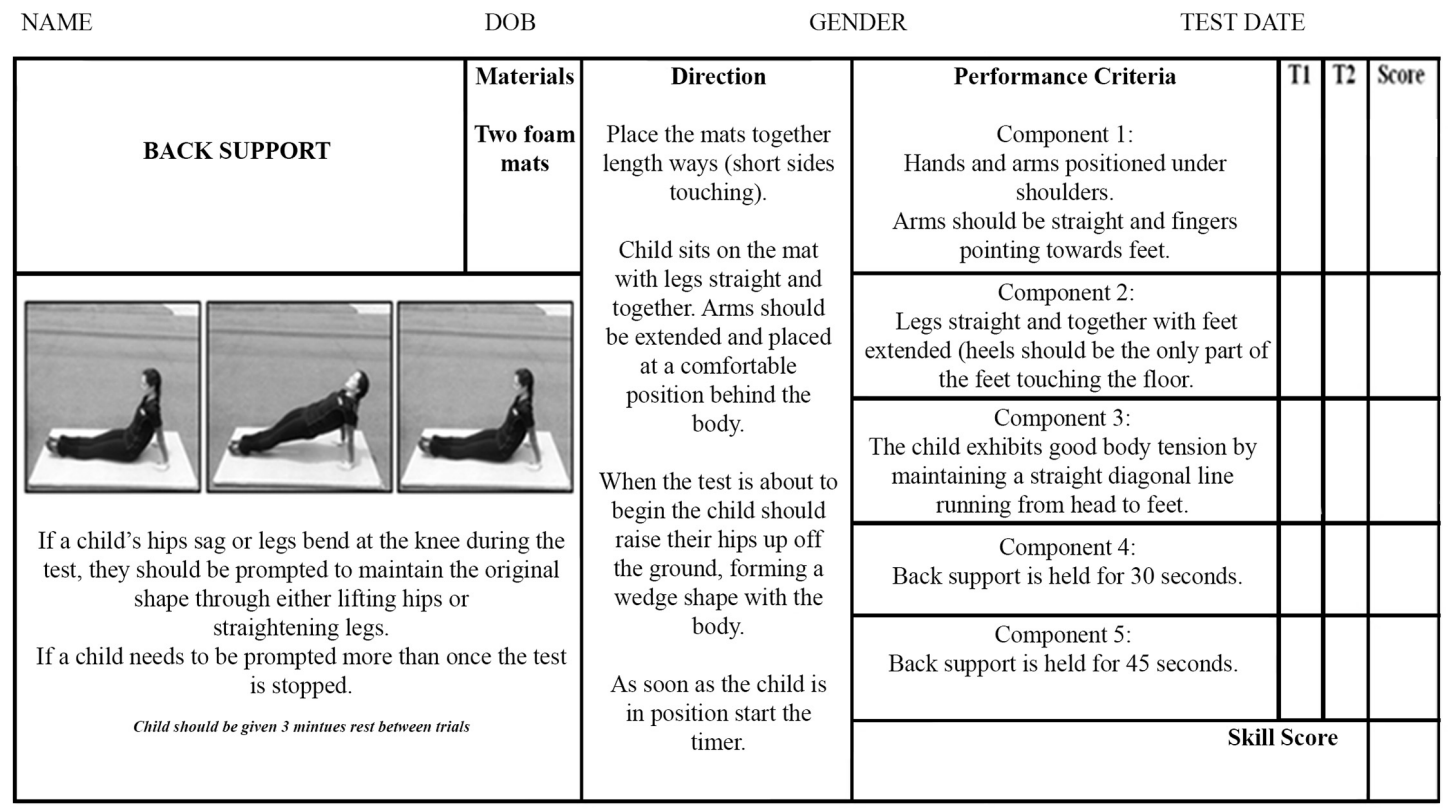
Back-support scoresheet.

Back Support: Feedback from the panel of experts resulted in the inclusion of two time based outcome components. In addition, successful completion of this task was deemed to include a high level of body stability as well as maintaining all-round body tension and strength. The new assessment break down was comprised of three process components and two timed product components (Fig 3). If a child was unable to maintain any of the process components (1–3) they would be given one prompt to re-hold the correct position, if they failed to maintain that position for a second time the test would be terminated. Alternatively, the test would be ended if the participant held the position for 45 seconds.

### Part Two: predictive validity and inter and retest reliability

#### Participants

Assessments to test the predictive validity of the stability skills involved a total of 337 children aged 6–10 (*M* age = 8.2 SD 1.2). We tested predictive validity in both gymnasts and children of differing SES backgrounds. To ensure a representative sample of children, the Socio-Economic Indexes for Areas (SEIFA) Index of Relative Socio-economic Advantage and Disadvantage developed by the Australian Bureau of Statistics (ABS) was used to identify a diverse selection of three schools. Overall, children were drawn from four cohorts; 37 (11%) gymnasts of mixed SES, 108 (32%) children from a high SES school, 128 (38%) medium SES school and 64 (19%) low SES school.

In order to assess the construct validity, confirmatory factor analysis was undertaken on the school sample only. This included a total of 300 school children (*M* age = 8.2, SD 1.1), of whom 155 (52%) were boys and 145 (48%) were girls. The school groups were assessed on the postural skills and FMS (Test of Gross Movement Development-2). Victoria University Human Research Ethics Committee (VUHREC) and Victorian Department of Education and Early Childhood Development approved the study and written parental consent was obtained for all participants along with participants assent to participate.

#### Assessment tools

Height was assessed with a Mentone PE087 portable stadiometer (Mentone Educational Centre, Melbourne, Australia) and weight was assessed using a SECA 761 balance scale (SECA GmbH & Co. KG., Birmingham, UK). To ensure reliability, two measures were taken and the average of the two was used. Body mass index (BMI) was calculated as weight divided by height squared (kg/m2)

An isometric handgrip dynamometer (TTM Dynamometer, Tsutsumi, Tokyo) was used as a measure of muscular strength. Measurements were repeated two times on the child’s dominant hand, the two trials were conducted with a pause of 30 seconds to avoid muscle fatigue. The result of each trial was recorded to the nearest 0.1 kg kilogram. If the difference between the two trials was within 0.5 kg, the test was complete. If the difference was greater than 0.5 kg, then we repeated the test once more after a 30 seconds rest period. Maximum score of the dominant hand was used in this study.

The Test of Gross Motor Development-2 (TGMD-2) (24) assesses proficiency in six locomotor skills (run, hop, slide, gallop, leap, and horizontal jump), and six object control skills (striking a stationary ball, stationary dribble, catch, kick, overhand throw, and underhand roll). Each participant completes all 12 skills of the TGMD-2. For each skill, components are marked as ‘present’ or ‘absent’

To measure stability, three additional gymnastics training skills were assessed. These were the rock (Fig 1), log- roll (Fig 2) and back support (Fig 3).

#### Procedure for data collection

The full test battery comprised the stability skills and TGMD-2. The movement competency assessments were carried out in a large sports hall with groups of four participants rotating around three skill stations and one anthropometric station. The TGMD-2 was split between two stations, a locomotor skills station (run, hop, slide, gallop, leap, horizontal jump) and an object control skills station (striking a stationary ball, stationary dribble, catch, kick, overhand throw, and underhand roll). The three stability skills (rock, log roll, and back support) made up the third skill station. Before the start of each skill children watched a live and pre-recorded demonstration. Following this they were given one practice attempt and two assessment trials for each skill as per TGMD-2 protocol.

#### Inter and retest reliability

Before testing could be completed, four research assistants (RAs) each undertook 26 hours of reliability training. RA1 and RA2 were trained to code each of the 12 TGMD-2 skills and each scored 15 pre-recorded videos of children (5% of the total sample). RA3 and RA4 were trained to assess the three stability skills and scored 25 pre-recorded videos of children completing the three stability skills (8% of total sample). Retest reliability was assessed for the stability skills through the level of agreement of a single observer over a seven day period (ICC values < 0.4 were rated as poor, > 0.4 to 0.8 as moderate and > 0.8 as excellent) [25].

#### Statistical analysis

Raw mean descriptive results were reported for the stability skills and TGMD-2 tests for each cohort. Prior to statistical analysis, the stability skills and TGMD-2 data were z-transformed. In addition, data was assessed for violation of the assumptions of normality and for outliers. We also examined the proportion of participants who scored towards the top (ceiling effect) or bottom (floor effect) of the test by examining the percentage of children who scored zero or a maximal score on the three stability skills.

To examine the predictive validity, the cohort differences were investigated. Analysis of covariance (ANCOVA) was conducted for the combined score of the three stability skills and controlling for the potentially confounding factors of BMI and grip- strength [17,18]. Multivariate analysis of covariance (MANCOVA) was conducted for the 3 stability skills separately with follow-up ANCOVAs in the instance of a significant main effect. Post-hoc comparisons were conducted using Bonferroni. Significance level was set at 0.05 and partial effect sizes were reported.

### Part 3: assessing how stability fits into a FMS Model

#### Confirmatory factor analysis

Confirmatory factor analysis (CFA) in AMOS 22 was used to examine the factorial structure of the three stability skills and if they loaded onto a single construct, named stability skills. CFA was conducted with the maximum likelihood method of estimation. In order to specify a model containing latent variables for all factors, error variance was set at zero. Several goodness of fit measures were used to describe the models. In addition to the Chi square (χ^2^) statistic, which is influenced by sample size and as such can be unreliable [26], the following fit indices were considered: Chi square/DF (X^2^/DF); Comparative fit index (CFI) [27]; Root mean square error of approximation [28]; Standardised root mean residual (SRMR) [29]; and the PCLOSE [30].

The χ^2^ statistic is a measure of overall fit of the model to the data with a non-significant P-value (P > .05) indicating a good fit. Also, χ^2^ divided by the degrees of freedom (χ^2^/df) provides an indicator of fit with values of < 2 considered adequate fit. CFI values of .90 or above indicate an adequate fit. RMSEA values of .06 or lower and SRMR values of .08 or lower indicate a close fit when these statistics are taken together. Finally, the PCLOSE should be non-significant (*p* > .05) [31,27].

#### Model specification

First the original FMS model comprised of locomotive and object control skills [32] was tested with the current cohort. In the instance of an adequate fit in both the stability skills CFA and FMS CFA, the new extended model of FMS would be tested; this would be comprised of stability skills, locomotive skills and object control skills.

## Results

### Descriptive data

Mean scores and standard deviations for children’s anthropometric, locomotor, object control and stability skills for the four cohorts are reported in Table 1. The test for multivariate kurtosis did not show problematic levels of skewness or kurtosis [33].

**Table 1:** Descriptive statistics [Means and standard deviations (M ± SD)] of Anthropometrics and aspects of Movement Competency for each cohort.

	Gymnasts (mixed SES)	School High SES	School Middle SES	School Low SES
N	37	108	128	64
BMI	16.1 ± 2.7	16.9 ± 2.6	16.9 ± 3.2	17.6 ± 3.0
Grip Strength	14.1 ± 5.1	15.5 ± 3.7	15.2 ± 3.4	14.8 ± 4.5
Stability Skills	21.5 ± 2.2	15.0 ± 4.9	12.9 ± 4.4	9.7 ± 4.5
Locomotive	32.0 ± 6.8	30.5 ± 7.3	28.6 ± 5.9	29.5 ± 6.5
Object Control	29.5 ± 8.2	30.7 ± 7.4	26.8 ± 8.3	30.3 ± 7.6

### Stability skills feasibility

The log roll had the largest floor effect with 29% scoring zero. The other two skills had 3% and 2% of children scoring zero for the rock and back support respectively. The back support and rock showed the largest ceiling effect with 25% and 22% of children respectively achieving a maximal score followed by 6% for the log roll.

### Inter and test re-test reliability

The Intra Class Correlations (ICC) for inter-rater reliability were all good: locomotor skills (ICC = 0.90; 95% CI: 0.73–0.98), object control skills (ICC = 0.82; 95% CI: 0.58–0.96), and rock: (ICC = 0.87; 95% CI: 0.73–0.94), log roll (ICC = 0.81; 95% CI: 0.52–0.93) and back support (ICC = 0.87; 95% CI: 0.72–0.95).

Test re-test reliability over a seven day period also demonstrated excellent consistency for each of the three skills: rock (ICC = 0.95; 95% CI: 0.83–0.98), log roll (ICC = 0.87; 95% CI: 0.59–0.95) and back support (ICC = 0 .88; 95% CI: 0.65–0.96).

### Predictive validity stability skills

Individual stability skills and total mean scores and standard deviations are reported in Table 1 for each of the four cohorts separately. ANCOVA for summed stability skills controlling for BMI and grip-strength showed a significant main effect (F (3.333) = 61.56; *p* = .001; η2 = .36). Post hoc comparisons revealed that all cohorts performed as expected with gymnasts (mixed SES) having superior stability skills than all other groups. Children from the high SES school scored better than the children from the mid and low SES schools and the children from the mid SES school scored better than the children from the low SES school.

**Fig 4:**
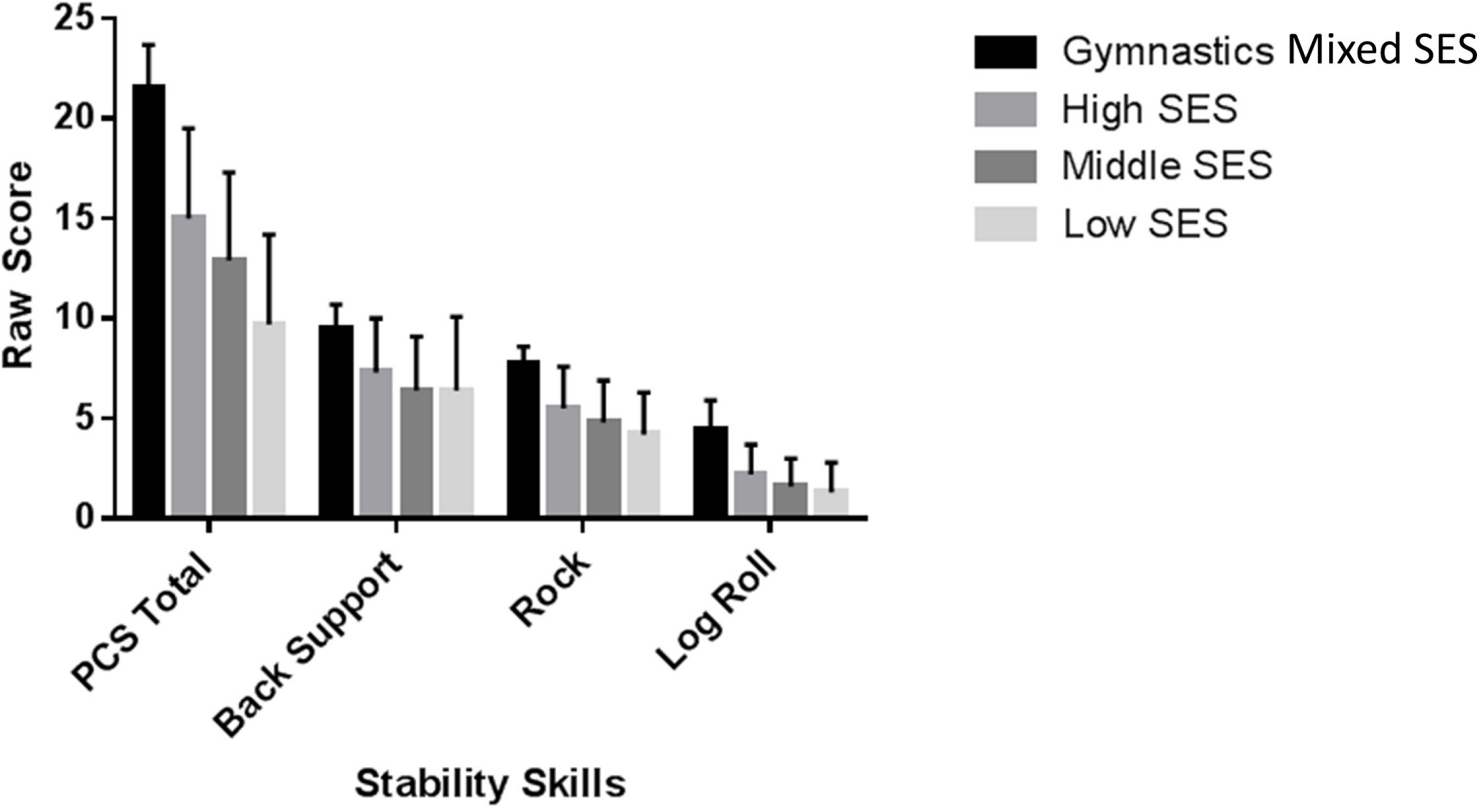
Mean scores and standard deviations for the four cohorts on the three stability skills.

MANCOVA for the three stability skills showed a significant main effect (Wilk’ λ = .61; F(3,9) = 20.67, *p* = .001; partial η2 = .16). Follow-up ANCOVA showed significant main effect for the rock (F(3,333) = 28.9, *p* = 0.01; partial η2 = .21), log-roll (F(3,333) = 32.65, *p* = .001; partial η2 = .23) and back-support (F(3,33) = 35.84, *p* = .001; partial η2 = .25). Gymnasts (mixed SES) scored significantly higher than all school cohorts on all of the stability skills. The high SES school cohort scored significantly better than low SES school cohort on all of the stability skills while the medium SES school cohort scored significantly better than the low SES school on back support and rock (all *p* < .05) (Fig 4).

BMI and grip-strength were significant covariates for all three skills, except log roll where BMI did not have a significant effect. Effects sizes for BMI ranged from 0.004–0.04 and for grip-strength between 0.02–0.11.

### Construct validity for the three stability skills

**Fig 5:**
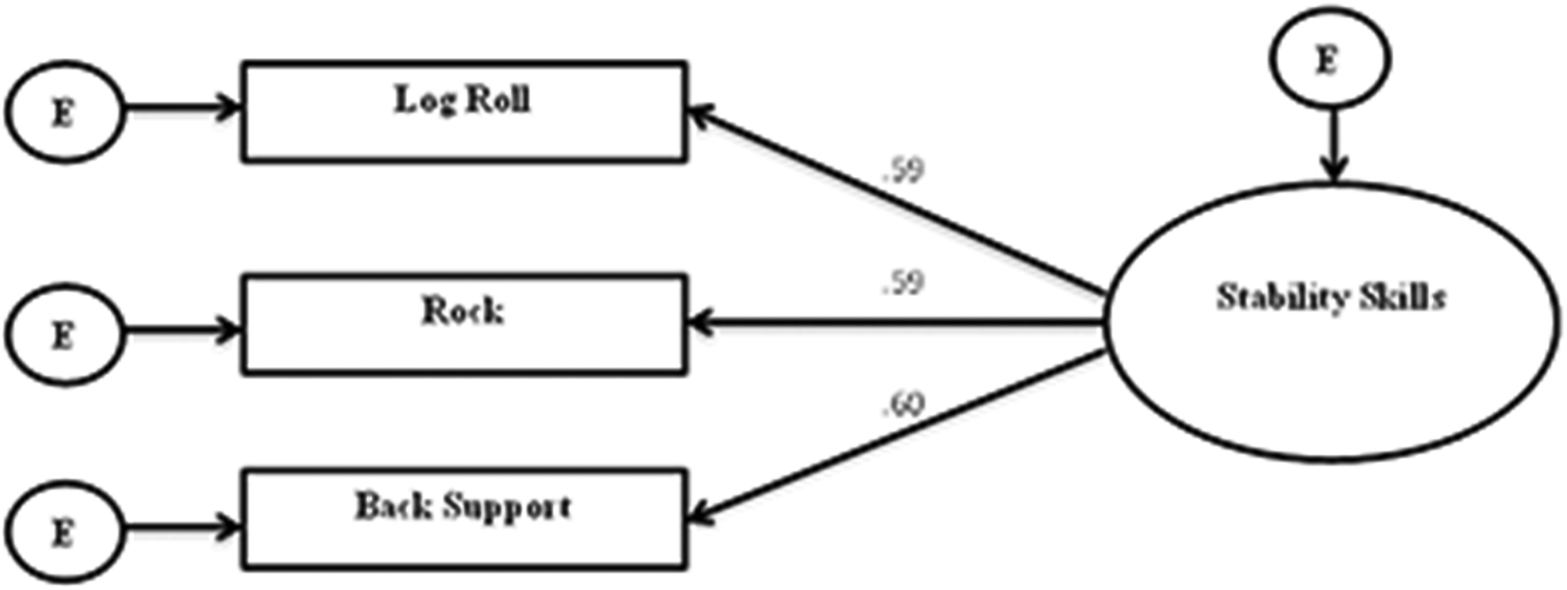
CFA for the three stability skills on the latent variable stability.

The Confirmatory Factor Analysis for the three stability skills (Fig 5) provided an adequate model fit (χ^2^ (2df) = 1.03; P = 0.6; χ^2^/df = 0.52; CFI = 1.00; SRMR = .02; RMSEA = .01; PCLOSE = .78). In this model the three skills had a moderate to strong effect on the latent variable stability skills (back support r = .60, rock r = .59, logroll r = .59). The total variance explained in stability skills was 56.5%.

### Construct validity of the Fundamental Movement Skill model

The original model of FMS was rebuilt according to Ulrich (24). CFA for locomotive skills demonstrated an adequate fit (χ^2^ (9df) = 10.8; P = .3; χ^2^/df = 1.2; CFI = .98; SRMR = .03; RMSEA = .03; PCLOSE = .76). The CFA for object control skills also demonstrated an adequate fit (χ^2^ (5df) = 3.70; P = .60; χ^2^/df = 0.76; CFI = 1.0; SRMR = .02; RMSEA = .001; PCLOSE = .86). These two constructs were then combined into a SEM model as proposed by Ulrich (24). This model showed an adequate fit (χ^2^ (48df) = 95.46; *p* = .01; χ^2^/df = 1.98; CFI = .90; SRMR = .06; RMSEA = .06; PCLOSE = .30). In this model both latent variables had high factor loadings (object control r = .75, locomotor r = .91) on FMS.

**Fig 6:**
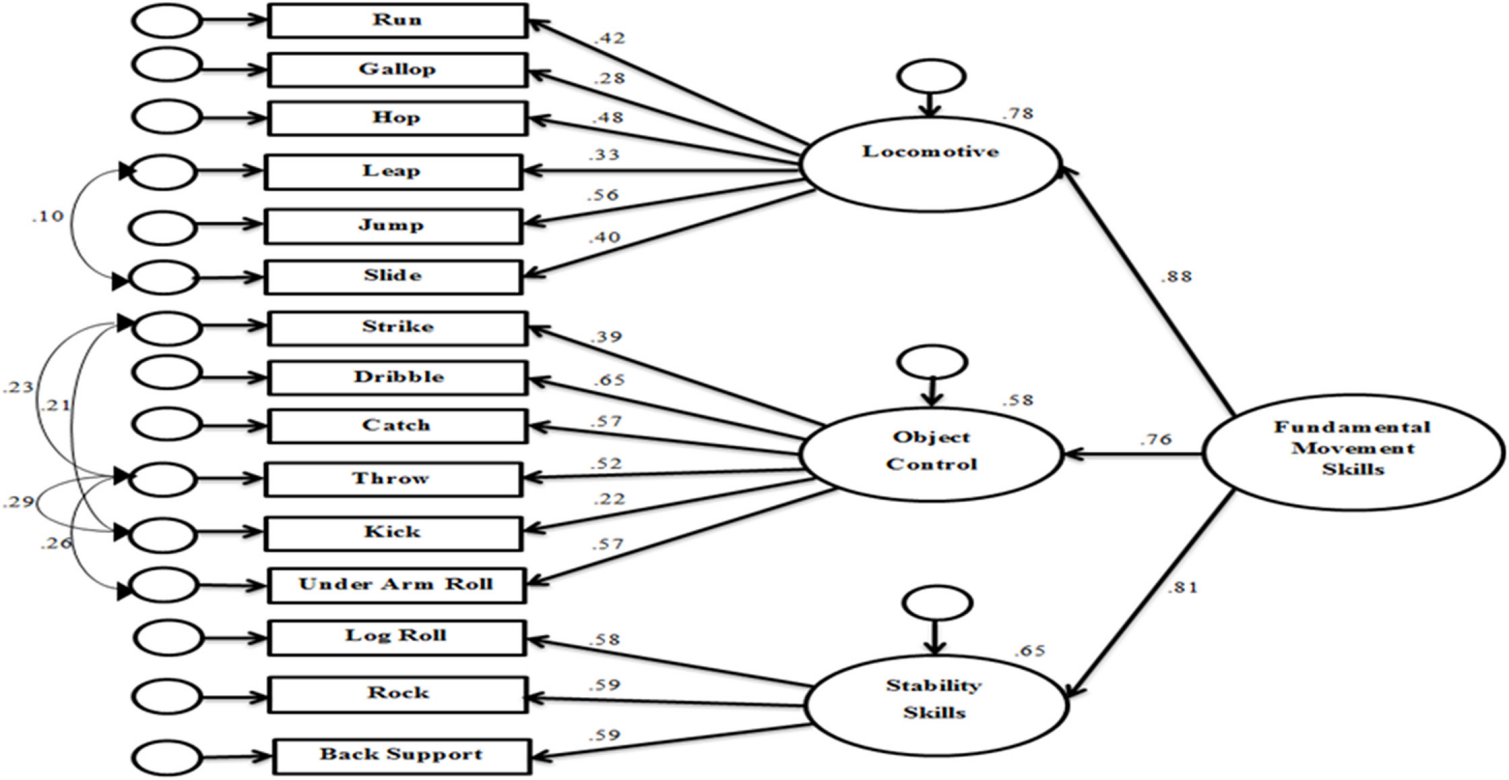
Complete model of Fundamental Movement Skills.

The final step was to combine the locomotor, object control and stability skills into a combined model of FMS. An adequate fit was achieved following some modifications (inclusion of correlating of error terms within individual factors) (χ^2^ (85) = 145.7; P = .001; χ^2^/df = 0.58; CFI = .91; SRMR = .06; RMSEA = .05; PCLOSE = .60). In this model locomotor (r = .88), object control (r = .76) and stability skills (r = .81) loaded on FMS (Fig 6).

## Discussion

This study aimed to a) develop a process based assessment tool to examine stability skills in children aged 6–10 years old; and, b) better understand the role of stability skills and their role in the development of FMS. A three-skill stability test battery was developed consisting of the rock, log-roll and back support task which had good face and content validity and inter rater and test-retest reliability. In addition, it was demonstrated that the individual skills, as well as the stability skills as a whole, had predictive and construct validity. Overall, the stability skills were found to be an independent factor in a FMS model and consequently they should be assessed separately to other facets of movement competency.

The systematic development of the stability skills test battery resulted in the selection of three gymnastics skills which can be used to examine children’s ability to orient and stabilize their bodies in space within a field setting. The rock and log roll were deemed to assess both orientation and stability while the back-support mainly assessed stability and torso strength. Using a mix of a process and outcome (back support only) assessment methodology the three skills collectively fitted well in a construct of defined stability skills (Fig 5).

Currently, there are limited process based tools in the motor development field to investigate the level of children’s stability skills in a school setting. This study has developed a process based assessment tool focused upon gymnastics skills as an alternative to the current product based assessment test batteries. By measuring the process/form of the movement rather than measuring the outcome of the skill it enables us to develop a comprehensive understanding of the strategies employed by children who have developed more efficient movement strategies in the stability skills domain. Indeed, empirical evidence suggests that there are associations between skill process and skill outcome [34,35].

The gymnasts outperformed the non-gymnasts in all skills. This finding is in line with Garcia et al. [21] research that participation in gymnastics develops superior stability skills through enhanced integration of where the body is in space during a task. The idea that stability skills can be accelerated through training and previous experience is further supported with school children from a high SES area outperforming school children from a medium SES and low SES area on stability skill performance. This is in line with previous research which shows children’s SES background impacts upon their maturational development [17,18], and the acquisition of FMS skills [19,20].

Overall, the children’s scores were distributed across the stability skills construct, although all were low compared to the gymnasts. The log roll appeared to be the most challenging skill for the children, as hypothesized during the validation stage, whilst the rock and back support were found to show ceiling effects. The three stability skills were successful in creating high postural demands on the postural control system leading to the development of an assessment battery which was able to differentiate across all children aged six to ten years showing superior sensitivity to its predecessors including the bipedial stance [21] and YBT-LQ [16]. Two potentially confounding factors, BMI and muscular strength, were found to have some (albeit, limited) effect on the sensitivity of the three stability skills showing the importance of adjusting for these factors. In general, the sensitivity to detect differences between SES backgrounds and gymnasts as well as finding good seven day retest and inter reliability between research assistants, provides a strong case that the stability skills tests would be able to pick up small changes as a result of intervention.

The second aim was to examine how the newly defined stability skills construct fitted into the FMS model. The original model by Ulrich [24] showed that locomotive and object control skills are measuring discrete constructs in a model of FMS. Our findings suggest that stability skills should be included into a model of FMS (Fig 6). The finding that the stability skills construct is largely discrete is an important finding and has consequences for development of test-batteries and the assessment of FMS/competence.

The results of this study suggest that a child’s stability skills will not reach their full potential by mainly focussing on practicing skills in object control and locomotor constructs as has been proposed in the academic literature [24,36,37]. Stability skills are better viewed as a separate construct that can be developed independently through a series of skills which challenge and place high demands on the postural control system. Appropriate practice would be gymnastics training or related whole body exercises that promote opportunities for children to rotate, invert and support their bodies using different body parts. These stability skills will place stress on the postural control system and result in children further developing sensory cues which will result in superior orientation and stabilisation strategies.

This new model of FMS where stability skills sit adjacent to locomotive and object control skills may be the result of changes in society which have created conditions where children’s basic skills are diminished compared to previous generations. Children now possess lower levels of movement competency, scoring poorly across the board, with low levels of object and locomotor skills [7,8]. The current study shows that the stability skills of children who have not experienced gymnastics training are poor compared to children who have. It is possible therefore that the decline is not only the result of children having decreased experience of incidental activity and spending more time indoors but is also due to the marginalisation of physical education in primary schools [38,39]. Educational gymnastics used to be a cornerstone of physical education in the Australian schools system [40], but that is now a distant memory as gymnastics teaching has declined due to an increased presence of sport education and sport pedagogy in which physical education has explicitly becomes focused on the development of skills required for team sports at the cost of perceived feminist sports such as gymnastics [41]. A recent article confirmed Australian children’s motor coordination was behind that of European counterparts who had gymnastics as part of their physical education program [42].

The strengths of this paper are the reporting of a reliable and valid instrument to assess stability skills. The process element of this tool gives instructors greater insights into children’s current movement strategies which will aid them in delivering quality feedback and to plan suitable interventions to improve stability skills.

This study has a number of limitations. First, the sample consisted of children from Australia only and therefore it should be explored if both this assessment and model can be generalized to children of other countries. Secondly, the rock and back support demonstrate ceiling effects with over one fifth of children scoring top marks for the assessments. The rock has already been refined in the content validation in an effort to enhance the orientation element and there may be little more that can be done. However, the back support task required participants to hold this position for 45 seconds and this could be extended. Future research should explore the relationship between the stability skills construct and how it correlates to other assessment tools which measure general coordination, rather than FMS, such as the Körperkoordinationstest für Kinder [43] which is popular in mainland Europe. Future research could also extend the measure of stability skills using the categorisation of stability skills as put forward by Gallahue et al. [1].

In conclusion, to date the stability skill construct has been poorly measured in field based movement competency research in children. This study provides a tool that teachers, practitioners and researchers can use to measure stability skills. This tool could be used alongside other FMS assessments to provide a better understanding of a child’s FMS development. In addition, our research suggests that stability skills can be viewed as an independent factor in a model of FMS.
